# Making things work–In spite of a pandemic small scale enterprise managers’ approach to business changes and health issues

**DOI:** 10.1371/journal.pone.0288837

**Published:** 2023-07-20

**Authors:** Bodil J. Landstad, Marianne Hedlund, Åsa Tjulin, Mikael Nordenmark, Stig Vinberg

**Affiliations:** 1 Faculty of Human Sciences, Mid Sweden University, Östersund, Sweden; 2 Unit of Research, Education and Development, Östersund Hospital, Östersund, Sweden; 3 Department of Social Work, Norwegian University of Science and Technology, Trondheim, Norway; 4 Department of Health Sciences, Mid Sweden University, Östersund, Sweden; The University of Queensland, AUSTRALIA

## Abstract

**Background:**

Covid-19 is one of the worst crises in modern working life with a direct negative impact on many enterprises and organizations. The aim of this study is to explore what managers in Small Scale Enterprises (SSEs) changed in their business during the Covid-19 pandemic, particularly addressing health issues.

**Methods:**

A longitudinal qualitative research methodology was used, interviewing 16 managers of SSEs in the Norwegian and Swedish manufacturing and service sector both before (round 1) and during the pandemic (round 2). In this approach, time is designed into the research process, making change a key focus for analysis.

**Results:**

The analysis resulted in two main themes, resilience and demanding occupational health and safety conditions, and five sub-themes. Results show how managers in SSEs changed their business during the Covid-19 pandemic and the impact of these changes. Although, the enterprises were heavily affected in the beginning of the pandemic, several managers found new solutions for their businesses to maintain and reach new customers. They applied a socially responsible management which addressed different health issues.

**Conclusion:**

Crises like the Covid-19 pandemic will have future impact on SSEs making it important to understand how managers in such enterprises address business and health issues. This knowledge may have practical implications for supporting managers in SSEs in how to perform a socially responsible management and maintain occupational health and safety measures. The managerial implications from this research are that they need to be flexible, reorientable and, at the same time, be loyal to the core business. This study shows the importance of doing longitudinal studies about business and health issues among mangers in SSEs.

## Introduction

Covid-19 is one of the worst crises in modern working life with a direct negative impact on many companies and organizations [1–5]. Until the pandemic, the Nordic work-life model has been given positive attention globally as the Nordic countries have shown good results in terms of growth, employment, gender equality, competitiveness, living conditions, and equality in relation to other countries [6]. The reason that is often cited is that the Nordic work-life model gives tools to deal with events such as economic and social downturns. However, the Covid-19 crisis hit the Nordic countries hard [5]. The aim of this study is to explore what managers in small scale enterprises (SSEs) changed in their business during the Covid-19 pandemic, particularly addressing health issues. By using interview data from two Nordic countries (Sweden and Norway) before and during the pandemic, this study addresses how the pandemic influenced these managers and what was essential for their health issues.

Research shows that occupational health and safety issues are less developed in SSEs compared to larger enterprises due to limited time and competence [7–9]. However, researchers argue that SSEs have organizational characteristics that are ideal for implementing working-condition improvements [10]. In a recent study [11], the findings showed that employees in small businesses reported better well-being during the pandemic if they perceived their employing business to have a strong safety and health climate. A study of enterprises in Sweden during the pandemic showed that SSEs applied hygiene measures to a higher degree, and social distancing measures to a lower degree, than larger enterprises [12].

The article is structured in the following way: first, research of relevance related to consequences of the pandemic for SSEs, prerequisites for the managers and a conceptual framework are presented. Thereafter follows the background to the study, the used materials and the method. In the result section, main findings are presented. Finally, the results are discussed in relation to other studies followed by conclusions, implications, strengths and limitations of the study.

### Consequences of the pandemic for SSEs

One group that face specific challenges is SSEs with less than 20 employees [11, 13–15]. They have fewer personnel and economical resources as well as a higher risk for income loss and may experience challenges working with occupational health and safety issues compared to larger enterprises [16, 17]. Exploration of how SSE managers and their enterprises have been affected by the pandemic is of great importance. SSEs are on the rise in many countries and are seen as key drivers of labor employment and economic development [18]. Before the Covid-19 pandemic, around 99 percent of the enterprises in Sweden were SSEs [19] whereas the corresponding figure for Norway was 98 percent [20].

In some countries there has been extensive governmental actions to assist the SSE managers during the Covid-19 pandemic. Income protection, expansion of paid sick leave and financial turnover programs have been main support measures [21]. Swedish support measures have been implemented by the central government to subsidize rent for those enterprises most affected by the crisis [21]. Other business support has included financial, in the form of loans, and managerial, in the form of advice and guidance for the start-up of SSEs [21]. Norwegian support measures have also been applied by the central government and included loans in crisis support for companies risking layoffs and bankruptcies as well as a major compensation scheme for businesses severely affected by the pandemic [22]. However, many of SSE managers did not apply for governmental support due to their uncertainty or perception that their situation does not meet the criteria and therefore are ineligible for support [1, 23].

### Pre-conditions for SSE managers

One can assume that working conditions and the well-being of SSE managers and their employees are more negatively affected by the pandemic compared to larger enterprises [14, 24]. This is problematic as research shows there is a relationship between financial hardship and impaired well-being among SSE managers [25]. In addition, manager’s well-being and working conditions are important, as shown in studies on relationships between well-being, and growth and sustainability of SSE businesses [26].

A study of French entrepreneurs concluded that the risks for burnout have increased so much during the pandemic making bankruptcy a predominant threat [26]. A study of the UK population showed that financial worries were associated with higher mental distress among the managers [27]. Extensive research points at the correlation of being an SSE manager with higher levels of uncertainty and responsibility in work tasks, intense time pressures and many working hours [28]. The work often consists of long and irregular working hours including evenings and weekends. However, SSE managers often have a high level of job control and freedom to determine which work tasks to implement and how to accomplish this [28, 29].

Leadership might influence employees’ health and there is support for positive effects of a health-promoting leadership [30, 31], which might be less developed in SSEs due to a lack of resources and incentives for addressing health issues [32, 33]. Reasons for this under-developed leadership could be minimal organizational mechanisms for communication, few in-house resources for occupational health issues, and a perceived lack of evidence for the benefits of workplace initiatives [34]. Health issues relate to culture in the organisation, and how organizations, managers and professionals try to influence employee’s ability to deal with health issues [35]. To obtain good business results, it is important to create a sustainable, healthy organizational culture [36]. Lopez [37] argues that managers in SSEs can influence the organizational culture by creating open communication and dialogue between managers and employees. Generally, it is important for managers in SSEs to promote employee well-being [38].

### Conceptual framework

According to Katare et al. [39], small businesses adjusted their operations to adapt to the changing times, such as altering customer service practices, procurement of supplies, and increasing advertisement in the form of social media presence during the pandemic. These changes were crucial for surviving the pandemic. According to Battisti & Deakins [40], a business’ dynamic capability to integrate resources in recognizing new opportunities is crucial in an environment characterized with high uncertainty.

Dynamic capabilities have been used in research to understand how businesses operate and can transform in conjunction with an ever-changing environment. The development of the concept dynamic capabilities has emerged primarily within management research where several definitions have evolved and are used in research in varied ways [41]. The original definition by Teece, Pisano, & Shuen [42]: *the firm’s ability to integrate*, *build*, *and reconfigure internal and external competences to address rapidly changing environments*, and the evolvement of the definition by Teece [43] that: *dynamic capabilities can be disaggregated into the capacity (a) to sense and shape opportunities and threats*, *(b) to seize opportunities*, *and (c) to maintain competitiveness through enhancing*, *combining*, *protecting*, *and*, *when necessary*, *reconfiguring the business enterprise’s intangible and tangible assets* can be useful in light of the Covid-19 pandemic.

To use dynamic capabilities as a conceptual framework could deepen the understanding of how SSEs adapted their work environment during the pandemic.

## Materials and methods

### Study design

The study uses a Longitudinal Qualitative Research (LQR) design. Interview data is collected in 2015 (round 1) and in 2021 (round 2). LQR is distinguished from other qualitative approaches by the way in which time is designed into the research process, making change a key focus for analysis [44]. As Thomson et al. [44] implies, this means that a re-study involves revisiting and updating an earlier study to discover new insights about the past in relation to the present day. Newly generated data are brought into dialogue with the findings and data from an earlier study, creating the means for a direct comparison between the two [45]. Neale [45] describes these studies as a sort of comparative research, but using comparisons over time, instead of over space.

### Recruitment

We recruited managers who took part in a previous project (2015–2017), inviting them to this new study in 2021. The managers were from different branches of SSEs from the private sector in Norway and Sweden. One selection criterion was that the participants should be managers of SSEs with less than 20 employees. Additional criteria were that they should be from rural areas (comparable geographic regions) and that they should represent different types of services in the private sector. The sampling was qualitative and purposive and was not aimed at serving representative purposes [46, 47]. The purposive selection was to interview a group of SSE managers. The sample differed internally, constituting a heterogeneous sample, but had the commonality of being managers in SSEs.

In round 1, 2015, eight managers in Norway and ten in Sweden were interviewed, and in round 2, 2021, sixteen of the same managers were interviewed, seven in Norway and nine in Sweden. Of the remaining two who did not participate in the later interviews, one had resigned as manager in 2021 and the other one withdrew from the study due to time constraints. Table 1 describes the characteristics of the sample.

**Table 1:** Characteristics and experiences of study participants (2015 and 2021).

	Round 1 (2015)		Round 2 (2021)	
Country	Norway	Sweden	Norway	Sweden
Managers in total	8	10	7	9
Gender				
• Men	4	6	4	5
• Women	4	4	3	4
Age				
• < 40	3	2	0	1
• 41–50	4	5	5	3
• 51–60	1	2	1	2
• > 61	0	1	1	3
Education				
• High school	0	2	0	1
• Vocational training school	2	2	2	2
• Upper secondary school	1	1	1	1
• University	5	5	4	5
Civil status				
• Married/cohabiting	5	8	5	7
• Single	3	2	2	2
Years in the enterprise				
• < 5	3	1	0	0
• 6–10	3	5	1	2
• > 11	2	4	6	7
Branches				
• Building & construction/industy	1	3	1	2
• Service delivery	7	7	6	7

### Data collection

The data collection period was between March and May 2015 in round 1 and between January and March 2021 in round 2. The data collection method in both 2015 and 2021 was focusing informant interviews [48]. The interviews lasted from 90–120 minutes in round 1 and from 15–90 minutes in round 2. In round 1 the interviews were conducted at locations convenient for the participants [47], and in round 2 the interviews were conducted via video link or telephone due to pandemic restrictions.

A semi-structured interview guide was used in both data collection rounds. In round 1 we asked for managers’ experiences and reflections on management policy, opportunities and obstacles for creating a healthy workplace, and the translation of working with occupational health and security issues in their enterprise. The interviews were replicated in round 2 with the addition of questions related to the Covid-19 situation. These questions touched on if and how the pandemic created new conditions and opportunities and if it also changed the existing conditions. We were interested in, as well, if the managers felt that new approaches implemented concerned health issues, and if the pandemic had any implications on work situation. In round 1 and 2 the tape-recorded interviews were transcribed immediately.

#### Data analyses

The data from both rounds (2105 and 2021) was compared and analysed to explore managers experiences and narratives, specifically in respect to changes before and after the Covid-19 pandemic. We analytically approached the fluid realm of temporality, however, the relationship between past, present, and future is complex. Time was analysed as a temporal gaze continually shifting as mangers look back and forth in the ever-moving present, overwriting SSEs development, reinterpreting wider social and structural forces. They emerge as interlocking flows of time that are in constant conversation with one another [49].

We analysed data from the two rounds into a comprehensive and coherent analysis of narratives about communication and changes. Analytically, we approached data about the changes and the content of these. We did not focus on the biographic development of every manager, but rather on their narratives and considerations. The main analytical focus was to explore what came into focus for the managers and what faded away as less essential for the development of the enterprise during the Covid-19.

The analytical steps included analysing the data from round 1 and round 2, and then compared them. The analysis was done by the first and the second authors, followed by a discussion of the results between all authors.

To obtain a sense of the whole interview, each interview was read in its entirety. To structure the analysis, the data were coded related to the two data rounds. When the data were coded in round 1 and round 2, we, alternated between the analysis of the two rounds. To collate the data into preliminary themes, we looked for patterns and diversity across the rounds. We used subthemes to structure the preliminary themes. The subthemes were abstracted into two main themes. The analytical outcome is illustrated in Table 2.

**Table 2:** Illustration of outcome of the analysis.

Theme	Sub-theme	Meaning units Round 1	Meaning units Round 2
Resilience	*Refinement of basic concept*	“Therefore, the advantages of a small company, when you own it … you can shape it … quite a lot yourself. You have this great power to make decisions … the opportunity. . . at the same time you have the disadvantages of the small company in that you don’t have the same. . . support functions in the company”. [IP 1]^1^	To maintain and ensure that we still have a customer base, there we felt quite immediately that we got away very lightly. Because. . . our customers are long-term customers. [IP 2]
	*Socially responsible management*	“… we try to sort out … not to sweep anything under the rug. If someone brings something up, we deal with it and try to solve it. Regardless of what it is. // … It is probably an important principle to demonstrate as a manager … To deal with things not like …”we will deal with that another day”, sort of “that was no big deal.” … That attitude is not like OK”. [IP 4]^2^	I mean, my employees, some of them have started building houses, some have bought new apartments. It’s a disaster, tragedies will happen if we don’t stand on our feet and move on. So, it’s just a matter of stepping forward and doing what needs to be done. There is not much to choose from. [IP10]
Demanding occupational health and safety conditions	*Adjustment of work culture*	“So not only should one work out, but that one meets others… to keep a good work environment is also a health-promoting thing. // Just do things together. Therefore, we have social gatherings besides working out, perhaps more unhealthy ones, perhaps a few beers on payday”. [Rn4]^3^	The work situation has changed. We can’t have our coffee breaks together anymore, because then there will be too many of us in the same place. We go for breaks in batches. So, the “being a team” and togetherness that we usually feel, for example at coffee breaks and other [social] events like this has been seriously affected. There will be groupings and distancing, that’s how it is. [IP10]
	*Applying regulations*	“The only thing I worry about … the work environment requirements and that kind of thing. // … that you are in the gray area of the law in some situations. // Of course, I am not on top of all that. Then you are going to be rapped over the knuckles sooner or later, when you haven’t met some requirement or you don’t have some document that is supposed to be in place”.[IP 4]^1^	We have since given the restriction advice, that you cannot come here if you have a cold. It was a little challenging at the beginning with some who. . . Yes, you know, you come to work anyway, that’s the mentality you’ve had. But now I think the token has fallen. . . as we can now refer to the pandemic law and say that if you come to work against the policy, we can actually report you to the Swedish Environment Agency and say that there has been an incident here. [IP3]
	*Creating a cohesive work culture*	“I think those who work here, they have a feeling of working together, in a team and helping each other. The feedback I receive is that largely it is the case that they … if someone is working on something and someone else is free, they come and help”. [IP 16]^2^	Yes, so we have had more meetings in 2020 than we have ever had. It’s because it’s so easy, Teams-meetings and so on. ’We take an hour now and update each other and other things’, we have not done that before. It is almost as if we have perhaps become more efficient in certain matters and can make decisions more quickly. Instead of a month passing its ’yes, but now we’ll take a quick meet’. [IP7]

^1^Hedlund M, Landstad BJ, Vinberg S. Tightrope walking–the external context of workplace health management in small-scale enterprises–a case study in Norway and Sweden. *Society*, *Health & Vulnerability*, 2017.

^2^Landstad BJ, Hedlund M, Vinberg S. How managers of small-scale enterprises can create a health promoting corporate culture. *International Journal of Workplace Health Management*, 2017; 10(3), 1–23.

^3^Vinberg S, Hansen E, Hedlund M, Landstad BJ. Ambiguity among managers in small-scale enterprises: how to handle business as usual and workplace health management. *Societies*, November 2017.

### Ethical considerations

The Regional Ethical Committee, Department of Medical Research approved the study in 2015 (Dnr 2014-28-31M), and in 2021 The Ethics Review Authority (Dnr 2020–05223) approved the follow up study. The informants in both 2015 and 2021 gave written consent to participate in the study. The participants were informed about their option to withdraw from the study without giving reason. We immediately anonymized identifying data in the transcriptions of the interviews. All data were stored according to the Swedish Act on Ethical Review of Research Involving Humans (SFS 2003:460 [2005]).

## Results

Here we present the results from the analysis regarding how managers in SSEs changed their business during the Covid-19 pandemic. The results are presented in nuances and contrasts of mangers comprehension of more "normal circumstances" before the pandemic (in round 1) versus how the situation became during the pandemic (in round 2). Emphasis is made on critical events that influenced the managers understanding of the SSE during the pandemic. The analysis showed two main themes: (1) Resilience and (2) Demanding occupational health and safety conditions. In the theme Resilience two sub-themes emerged: *Refinement of the basic concept*, and *Social responsible management*. In the theme Demanding occupational health and safety conditions three sub-themes emerged: *Adjustment of work culture*, *Applying regulations* and *Creating a cohesive work culture*.

### Resilience

Being a manager in a SSE involves personal engagement and dedication to keep the business going despite challenges. These are qualities that managers emphasise particularly in round 2, during the pandemic. The ability to recover in times of crises and when the business is bent and stretched is evident for the resilience the managers demonstrated.

#### Refinement of the basic concept

In round 2 the managers described the experience of first two weeks of the March 2020 lock down as being in a vacuum: the inbox was empty and the phone silent, i.e. no business was coming in to the enterprise. After these two weeks they started to reorientate and act. Things needed to get back to normal, and the managers began to reflect how they could get started in business again. From being a successful business in round 1, to being ‘nothing’ felt dramatic for the managers. They needed to consider their options and implement a plan to appeal to customers. Finding new ways to deliver services and keep a position in the market became critical. They also underlined the importance of establishing hope for the future and the ability to survive. For some, the business went better than ever.

I have come out of it extremely lightly. Last year was the best year overall since I started as an entrepreneur. It has been a great year. // It was an absolutely superb year last year. So no, I can’t say that I’ve lost anything from corona, it’s almost the opposite. We received some compensation from employer contributions last year and spring and… Yes, it came automatically, so there was money in the bank immediately. It was almost an advantage for us with corona, if I’m being really honest. [IP5]

The managers of successful businesses made efforts to maintain customer relations and ensure that a customer base was kept during the pandemic in round 2. This was important for the survival of the enterprise and to maintain a normal existence and autonomy in the market. The customer base was a result of consistent work over time for the managers in SSEs, and therefore they wanted to show loyalty and appreciation. When the pandemic occurred, these managers realized the importance of going back to basic concepts overhaul of their products/services.

To maintain and ensure that we still have a customer base, there we felt quite immediately that we got away very lightly. Because… our customers are long-term customers. [IP 2]

In round 1, before the pandemic outbreak, “normal” was constantly evolving and renewing the enterprise, i.e., to develop new services and products. However, in round 2, after the outbreak, it became important for managers to return to basics and the core of the business. They appraised the core business as essential for keeping their head above water in a struggle to hold the market position. In this way the core business represented a normality they strived towards. One example was a contractor who went back to the main concept in traditional building of houses instead of trying to satisfy new customer groups with more demanding and advanced building technics as this required closer physical collaboration with customers, which they considered risky at the beginning of the pandemic.

In round 2, at the start of the pandemic, managers dedicated a lot of time to orient themselves in the chaotic situation. Reviewing the information from public announcements and assessing the implications for the SSE became the priority. As the pandemic continued and became more of the norm, this priority smoothed out as they acquainted themselves to the current situation.

In the beginning, there was very little information… Or, then we didn’t really know where to look, and there were 100 different sources, and it was difficult. I listened to press conferences and watched the news and stuff like that, but now it’s a little calmer on that front. Now we know how and where to search. [IP3]

The managers strived to maintain a kind of everyday life for the SSE and the employees. Despite a very unnormal and chaotic situation in round 2, it was fundamental for them to get ‘back to normal’. This was expressed by one manager to mean keeping the services as is, as it was a proven solution. During the first period of the pandemic, they had developed a model for resilience so they could be helpful to the customers regardless of challenges in time and space.

#### Socially responsible management

The importance for the managers to maintain responsible management was elevated due to the pandemic. It was important prior to the pandemic in round 1, and in round 2, even more. Managers felt responsible for the enterprise and their employee’s future employment expressing a social responsibility towards personnel for a fulfilment of economic commitment. This was perceived as a vital task both for keeping the SSE going and to maintain jobs in the region, especially since some SSEs where enterprises in sparsely populated areas, meaning their employees could not easily find other forms of employment, if needed.

I mean, my employees, some of them have started building houses, some have bought new apartments. It’s a disaster, tragedies will happen if we don’t stand on our feet and move on. So, it’s just a matter of stepping forward and doing what needs to be done. There is not much to choose from. [IP10]

Managers in SSEs expressed how important it was to support and sponsor leisure activities in the local community. This was a responsibility they felt before the pandemic in round 1, which became even more important in round 2, during the pandemic. During crises, these activities could become vulnerable, and need financial support. When business was going well, they could provide financial support that helped keeping the activities in the local community going. In this way managers in SSEs expressed a need to operate in a socially responsible way for their local communities.

But then, one more thing that has… well, in the pandemic that is. All sports clubs and everything, it has become that they have gone in and sponsored almost ten times more during the year compared to previous years. // It has been going well for us, and they’ve been struggling. And then I think, then it feels like the time to… well, it has for a long time now, been the right time to support more. // You get it in the end, that’s how it is. [IP5]

Managers would show social responsibility to customers even if they could not use the services of the enterprise. For example, in round 2, during the pandemic, preschools in Norway were closed to non-essential worker-families. In this instance, in a co-op preschool, the manager tried to support the families who were required to keep their children home by offering activities bags with borrowed toys or games to activate the children they had at home.

We got to spend a lot of time in contact with the homes then. Called around and talked to them and arranged some activity bags for the children who were not in the preschool. The parents came and collected these activity bags. There were slightly different tasks then. It was really quite demanding as a manager. Because suddenly I had tasks pulling me in many different directions. [IP11]

In another privately own preschool, the managers expressed a concern for children with special needs. They felt a social responsibility for these families, and therefore paid particular attention to them, keeping in contact and checking up on the families at home, ask how the children were doing and offering assistance. In that sense, the managers revealed socially responsible management in the sense that they scrupulously cared for needs of their customers. Social responsibility was vital for managers before the pandemic in round 1, but in round 2, during the pandemic socially responsible management description changed context. The services of the SSEs became more meaningful, and managers increased their social engagement with fulfilling needs of the customers and community.

### Demanding occupational health and safety conditions

Managers of SSE experienced demanding conditions in round 2 for carrying out occupational health and safety issues during the pandemic. Prior to the pandemic in round 1, managers could experience occupational health and safety as challenging to carry out. This became even more demanding and important in round 2, during the outbreak of the pandemic. Any workplace needed to pay attention to these issues if they wanted to keep their business going.

Managers needed to adjust to new solutions for working under new conditions. They needed to face whatever challenge occurred and try to promote an acceptable healthy workplace and simultaneously follow regulations given by the public health authorities.

#### Adjustment of work culture

In round 1 it was easier for managers to develop a good work culture through gathering employees. Shortly after the outbreak of the Covid-19 pandemic, new recommendations were introduced for social distancing. This meant regulations and recommendations about staying at home, limiting travel, avoiding crowded areas, using no-contact greetings, and physically distance from non-family members. In Norway these became mandatory by government, while in Sweden social distancing was a recommendation. This affected the SSEs and their managers in round 2: personal interaction in the workplace and new hygiene policies became the focus to decrease the risk for employees becoming infected with Covid-19.

The work situation has changed. We can’t have our coffee breaks together anymore, because then there will be too many of us in the same place. We go for breaks in batches. So, the “being a team” and togetherness that we usually feel, for example at coffee breaks and other [social] events like this has been seriously affected. There will be groupings and distancing, that’s how it is. [IP10]

In round 2 the managers felt vulnerable because of the low staffing numbers, who relied on each other. Many of the businesses offered specialized services which required a particular competence. This motived them to try to keep everyone healthy during the pandemic.

At the beginning, I thought everyone had a slightly calmer attitude, and then… just like that, someone knows someone who has died in the hospital, then it’s serious. Then you get worried. // So that now we don’t receive visitors from the outside, instead we stay in our big house alone keeping a good distance from each other… So I think everyone feels safe in the work environment. [IP3]

At some workplaces the employees were divided into cohorts and were instructed to keep a physical distance and only interact with others within the same cohort minimizing interactions. The manager was responsible for dividing the employees into cohorts and for ensuring that the rules were adhered to in order to follow the state outlined recommendations. This was a peculiar situation for the managers in round 2, who needed to think differently to secure a safe workplace culture.

Most of the time there has only been the two of us in the office and we have defined ourselves as close contacts and have had a working day that has been quite similar to the previous day. We have tried to be a little restrictive with customers coming to visit and so on, and so it has not been a matter of course that they have been invited in and given coffee. It could be that instead we have a chat out in the courtyard or up in the workshop. Because it’s a little easier to keep your distance, yes. Some things like that. [IP16]

As the virus is invisible there was a lot of uncertainty around everyday activities in round 2, for example, the handling of packages, especially from overseas. The question of which measures to implement to adhere to the recommendations and the keep the employees safe was not a clear conclusion and therefore weighed heavily on the managers. How far should the measures be taken: physically separating employees from customers, separating chairs distancing people, sanitizing packages before handling. “Have we done enough?”, was a question that weighed heavily on the managers in round 2.

In the beginning, it was not known whether the virus, for example, lived on packaging when a package arrived from abroad or something like that. You don’t know that. And I don’t know if everyone knows everything yet. So, then we had to think and every day you have to ask yourself… yes, have you thought about it, thought about keeping your distance? We moved the chairs and now it’s like in everyday life, we have all the time… we have introduced cleaning every day, to eliminate the risk of infection. And right now we have major cleaning going on because… because the branch organization has demanded it. [IP6]

#### Applying regulations

The focus in round 2, was on how to deal with anxiety and insecurity about the future. This differed in round 1, when safety issues at the workplace were more predictable. In the beginning, the pandemic was seen as a temporary situation, but as time passed, it became a persisting one which managers of the SSE needed to address daily, with each new wave of Covid-19.

Now that the so-called third wave is here, I think we are all a little more jumpy or a little afraid or more sensitive. So that the general situation is a little more uncertain and we talk about it every day—at coffee in the morning and then at lunch and so on.… I think that everyone vents what they feel in that case, they do. [IP3]

The pandemic situation meant new rules, recommendations, and regulations all the time for the SSEs in round 2. The managers were bombarded daily with new or updated regulations and recommendations. Their main task was determining which of the recommendations applied to their business’ situation, implementing them in a timely and effective method, keeping on top of business and trying to maintain a safe workplace. Norms and policies were put in place for when employees should stay at home and when they could come to work. Before the pandemic, in round 1, the work attitude was that one goes to work even though they have minimal symptoms, a runny nose or a slight cough, for example. The mentality was that these minor symptoms of colds: headaches, sneezing, cough etc. were no excuse to stay at home. With the pandemic, this all changed. The workplace, in round 2, became a risk arena for spreading the Covid-19 virus, and precautions needed to be taken.

We have since given the restriction advice, that you cannot come here if you have a cold. It was a little challenging at the beginning with some who… Yes, you know, you come to work anyway, that’s the mentality you’ve had. But now I think the token has fallen… as we can now refer to the pandemic law and say that if you come to work against the policy, we can actually report you to the Swedish Environment Agency and say that there has been an incident here. [IP3]

Round 2 showed, as well, an increased need for precaution when working outside the enterprise with clients and customers. The managers relied on their employees to be careful and keep social distancing. One manager of an SSE which serviced equipment for health care institutions, explained it the following way:

We were very conscious of this with close contacts, so limit it as much as possible. If we are to enter the institution buildings, then we are dependent on their trust in relation to us handling this with infection and close contacts and all that in a proper way. And that meant that in some contexts we also had to separate the employees. Otherwise, we became very vulnerable, because if one of them got infected, we would sort of have to quarantine the whole gang and that… yes… And we managed to avoid that then. [IP18]

Addressing the risk for being infected by Covid-19 became a task managers needed to pay attention to in round 2. They experienced that the employees were anxious and tense for catching the virus. Managers, for this reason, focused on preserving the psychosocial work environment.

Yes, we have not needed to have contact, but on the other hand, we have offered the staff, of course, from time to time… Or, they know that if they need to talk to a behavioral therapist or someone else then… other services are available, they can contact me and I will book it. But, no one has needed it so far, but we have been clear that this therapy is available. [IP3]

#### Creating a cohesive work culture

In round 2, working digitally and having online meetings became common in all SSEs. Particularly, in the beginning of the pandemic where restrictions existed for travelling and an exhortation to work at home for those who could. There came a need for managers and employees to communicate and participate in meetings without requiring a physical presence. The solution was to implement the use of digital platforms such as Zoom and Teams, making it an ordinary communication form within the business.

Yes, so we have had more meetings in 2020 than we have ever had. It’s because it’s so easy, Teams-meetings and so on. ’We take an hour now and update each other and other things’, we have not done that before. It is almost as if we have perhaps become more efficient in certain matters and can make decisions more quickly. Instead of a month passing its ’yes, but now we’ll take a quick meet’. [IP7]

Even though this new form of communication strengthens the flexibility of the business, as seen in round 2, as well as makes spontaneous communication easier due to eliminating the need for consider travelling to and from, etc., it also created new challenges. It became challenging as the employees did not have the same level of daily physical contact which made the workplace less cohesive. Previously, in round 1, when all work was done inhouse and employees were in the building, managers focused on creating a health-promoting corporate culture, characterized by inter-organizational dynamics and participative leadership. This was conditioned by the physical interactions of employees and managers resulting in a workplace characterized by corporate culture, solidarity, and flexibility. The pandemic effectively eliminated these interactions with the work-from-home policy. In round 2, the workplace suffered by losing its social dynamics as they had in round 1, regarding pattern of development and driving forces of a working group and sense of community. It became difficult to reestablish that same level of cohesive work culture and team atmosphere. The introduction of digital platforms, such as Teams and Zoom became tools for interaction and information sources. This made it easier to promote more cohesion at the workplace.

… now this with Teams has become very popular. We have become very good at digital meetings. // So this has become a small detail… which means that we have got more of that ’we-feeling’. Because now everyone in the organization knows what is going on at all times. Everyone gets information and everyone gives information away… so everyone gets the same information. So no one seems to get either more or less. [IP18]

The use of digital meetings was not limited to internal meetings. This digital tool became the go-to for all meetings in round 2. Managers of SSE have always found it useful to meet other managers in the same branches to discuss. Before the pandemic, in round 1, the meetings were physical, either by travelling together or by visiting each other’s locations, which due to the restrictions became impossible. Digital meetings have become the solution.

Cohesive work culture could also be stimulated by finding activities to do together despite restrictions. Instead of meeting at pub after work as they did in round 1, employees and managers could take a walk and make this a social event of importance to the workplace.

Not meeting at pub after work, but they’ve gone for a walk, yes. ‘Top 10’ [climbing mountains] is almost like that, some people become fanatical then, you know. The internal competition got so fierce that we had to cut it out, you know, because they got a little crazy. Got prolapsed and stretched and stuff like that. [IP13]

## Discussion

This study addresses how SSE managers changed their business during the Covid-19 pandemic, particularly as it concerns health issues. A longitudinal qualitative research methodology was used with interviews both before (round 1) and during the pandemic (round 2). SSEs in two sectors, manufacturing and service, in Norway and Sweden were studied. Results before the pandemic (round 1) showed how managers of SSEs can create a health promoting corporate culture [33]. Such culture is characterized by inter-organizational dynamics and participative leadership. Managers applied a process-oriented communication style, were all-rounders, and demonstrated dedicated and distinct management. In addition, they were restricted by financial limits, work environment and rehabilitation legislation, as well as heavy workloads, all the while being alone in the leader position.

Further results from round 1 demonstrated that managers had restricted leeway and commitments [32]. The findings explore which and how external factors impacted occupational health and safety issues. The SSEs had insufficient time and limited resources to address health and safety activities and insufficient knowledge about support systems. They pointed to a lack of competence and a high workload that hindered them. Managers experienced an ambiguity emerging from internal and external demands [50]. This pointed at challenges in dealing with many work tasks concerning financial decision-making, labour market legislation, staff development and maintaining business.

From the analyses two main themes evolved: (1) Resilience, and (2) Demanding occupational health and safety conditions. The managers demonstrated resilience, doing what was needed to keep the business from bankruptcy. In the beginning of the pandemic, the managers described the enterprise as heavily affected by the aftermath and response of the pandemic. The businesses needed to reorient their policies and find new solutions during the pandemic. This is in line with other studies that showed that many SSEs lost customers during the pandemic that led to reduced income and new burdens for the managers [2, 51]. However, it was noted in another study that SSEs using dynamic capability strategies became more effective compared to larger enterprises [2]. Dynamic capabilities were also important for the SSEs in the presented study. Dynamic capabilities gave the SSEs ability to integrate, build and reconfigure internal and external competences to address rapidly changing environments [2]. The SSEs in the present study showed an increased capability to address health issues in round 2.

The findings indicate that managers focus on their core business when crises such as a pandemic occurs. This is in line with Ozanne et al [52] research showing managers capability to respond, or seizing to the pandemic to cope and keep value for customers.

Socially responsible management was noticeable in round 1 and became even more noticeable in round 2. During the pandemic the managers increased their efforts to maintain jobs for the employee’s and to contribute to the local community. Managers valued their business’ importance to the community and wanted to remain an attractive workplace. These efforts and the growing interest for social responsibility of SSEs are underlined in other studies [53, 54]. The managers in the present study experienced demanding occupational health and safety conditions in round 1 and even more demanding in round 2. They needed to follow health authorities’ pandemic guidelines and rules in parallel to considering their employee’s health and security while on the job. Managers implemented social distancing within the business by dividing the employees into cohorts to reduce contact and therefore the risk of infection. One of the struggles the managers needed to attend to, was their employees’ persistent anxiety over fears of infection by the Covid-19 virus and how following health conditions would affect their families. Some employees belonged to a risk group or cohabitated with someone in a risk group for severe illness. This meant another level of awareness of a socially responsible management; managers needed to predict and develop tools for how to handle this fear among employees. Another Swedish interview study, demonstrated that organizations handling occupational health and safety risks with established routines were less affected by the pandemic as they could adjust work environment- and risk factors [55]. Organizations focusing on social and organizational work aspects needed to re-prioritize and focus more on the physical work environment and risk management [55].

A noticeably change between round 1 and 2 was that the SSEs in this study began to work more from home with online meetings for communication. This is in line with other research [56]. The managers and employees had to learn to use new digital platforms and to find other ways to communicate and marketing their services or products. In round 1 the managers focused on creating a cohesive work culture. In round 2, this culture was difficult to maintain. Other studies point at a changed leader role during the pandemic with increased areas of responsibilities, expectations, and requirements [15, 57]. In round 1, the managers found it useful to communicate in networks with other managers. This continued to be important in round 2, although most of the communication was done digitally.

In round 2, the managers appreciated the authorities’ guidelines for handling the pandemic. The guidelines facilitated how to address issues of safety and how to minimize the spread of the virus, therefore allowing managers to concentrate on the business. They had an unmet need for consultation on occupational health services, how to handle their employees’ as well as their own working conditions and well-being. This need was difficult to manage. Occupational health services could not provide this service due to restrictions on visiting workplaces to evaluate and propose measures.

A change in addressing health issues was that SSE managers became increasingly concerned with infection prevention and virus control in round 2. In round 1 the concern was more advising on work conditions generally and employees’ well-being, creating good conditions for employee’s health at work in a broader sense. The focus on health became narrower in round 2. Most attention was given health issues such as hygiene and infection prevention measures. Concerning psychosocial and physical working environment factors, the SSE managers experienced problems to prioritize measures related to these areas in round 2. These issues were more in focus in round 1. However, in round 2, managers focused on a safe culture and handling anxiety among employees, although this was challenging because the work became more and more digital with fewer physical contacts at the workplace.

## Conclusions

In this study we found that managers in SSEs changed their business during the Covid-19 pandemic. These changes addressed new challenges for addressing health issues. The managers needed to refine the basic concept, keep the business going, practice socially responsible management, and address demanding occupational health and safety issues. Social distancing became the norm in round 2 and managers needed to deal with persisting anxiety among employees and how to work differently but keeping a cohesive work culture. A conclusion is that SSE mangers used dynamic capability strategies to better transform and sustain their enterprises during the pandemic. This implies that the concept of dynamic capabilities is relevant when analyzing managers of SSEs regarding crisis as the Covid-19 pandemic.

The Covid-19 pandemic and other pandemic crises will probably continue to have an impact on managers and employees working conditions and well-being in the future. This makes it important to watch how managers in SSEs adapt to perform socially responsible and health promoting management. Knowing the needs of managers that may arise in such crises is advantageous for appropriate governmental and community support programs, legislation, and policy to ensure longevity and the ability of these enterprises to ride out the storm and come out on the other end. For example, managers may need to collaborate with occupational health services, other consultants, and other managers of SSEs in times of crises. Earlier research emphasizes the need for external support for occupational health and safety improvements [33, 50, 58]. Managerial implications are that it is important for the managers to apply a socially responsible management approach and to use elements related to dynamic capabilities. External support can also be collaboration with the academic sector, because research show that socially reputable companies have a high degree of links to academic institutions [59].

The implication of this study is that there is a need for deeper understanding of the dilemmas managers face regarding how to approach health issues in SSEs, particularly when crises occur. Managers need to implement changes to business practices including health issues. Understanding that the practice of managers in SSEs could be studied from theoretical angles of their dynamic capabilities’ strategies. This would give us insight into how they resolve dilemmas in the daily life of their business. The managerial implications from this research shows that managers need to be flexible, reorientable and, at the same time, loyal to the core business.

Decisions made by policy makers need to take into consideration the long-term impact of the pandemic on SSEs, their managers and employee’s. Our study shows the importance of doing longitudinal studies about business and health issues among mangers in SSEs.

### Strengths and limitations

A strength of this study is that it included mangers from different types of SSE sectors with various experiences. It is also a strength that the extensive interviews at two different times with the same managers made it possible to deepen the understanding of the pandemic’s effect on small business managers in their specific contexts. This made a longitudinal qualitative methodology possible.

There are both strengths and weakness using such approach. A benefit, growing from the quality of a continuous research relationship, is the possibility to incorporate changes into the analysis. By bringing researchers’ reflections on the interview encounter into the data through records of their hopes, fears, predictions, we could bring interactive and formal aspects of their experience within the frame of analysis. The data analysis and data management were highly labour intensive, which is seen as a weakness to the method. An analysis of the data on both cross-sectional and temporal dimensions [60] was required.

To ensure that results are dependable and confirmable, notes were taken on decisions made during the entire research process, inclusive reflective thoughts after interviews, sampling and research materials adopted were saved in order to be able to review the transparency of the research path [61]. A strength and limitation with this analytical approach is that the interpretations are always provisional and difficult to manifest. A continuity of researchers’ observations and interpretation of the interviews enabled the researchers to shift from provisional interpretations and to clear interpretative outcomes.

Another limitation to the study is that the results were confined to the original participants of round 1 in the study. These small business managers were not specifically chosen based on enterprises strongly affected by the Covid-19 pandemic, e.g., tourism, hospitality and restaurant, entertainment, and air transport industry. It is unknown if the results are transferrable to other effected branches. Another limitation is that data was primarily gathered in a single geographical area of Sweden and Norway and not representative for all SSEs nor regions. It is therefore uncertain if the results are transferable.
